# Neurochemical Changes Associated with Stress-Induced Sleep Disturbance in Rats: *In Vivo* and *In Vitro* Measurements

**DOI:** 10.1371/journal.pone.0153346

**Published:** 2016-04-14

**Authors:** Do-Wan Lee, Seockhoon Chung, Hyun Ju Yoo, Su Jung Kim, Chul-Woong Woo, Sang-Tae Kim, Dong-Hoon Lee, Kyung Won Kim, Jeong-Kon Kim, Jin Seong Lee, Choong Gon Choi, Woo Hyun Shim, Yoonseok Choi, Dong-Cheol Woo

**Affiliations:** 1 Division of MR Research, Department of Radiology, Johns Hopkins University School of Medicine, Baltimore, Maryland, United States of America; 2 MR Core Laboratory, Asan Institute for Life Sciences, Asan Medical Center, Seoul, Republic of Korea; 3 Department of Psychiatry, Asan Medical Center, University of Ulsan College of Medicine, Seoul, Republic of Korea; 4 Biomedical Research Center, Asan Institute for Life Sciences, Asan Medical Center, University of Ulsan College of Medicine, Seoul, Republic of Korea; 5 Department of Radiology, Asan Medical Center, University of Ulsan College of Medicine, Seoul, Republic of Korea; Radboud University Medical Centre, NETHERLANDS

## Abstract

The goal of this study was to quantitatively assess the changes in the cerebral neurochemical profile and to identify those factors that contribute to the alteration of endogenous biomolecules when rats are subjected to stress-induced sleep disturbance. We exposed Sprague-Dawley rats (controls: *n* = 9; stress-induced sleep perturbation rats: *n* = 11) to a psychological stressor (cage exchange method) to achieve stress-induced sleep perturbation. *In vivo* magnetic resonance imaging assessments were carried out using a high-resolution 9.4 T system. For *in vivo* neurochemical analysis, a single voxel was localized in the right dorsal hippocampal region, and *in vivo* spectra were quantified for 17 cerebral neurochemical signals. Rats were sacrificed upon completion of the magnetic resonance spectroscopy protocol, and whole-brain tissue was harvested from twenty subjects. The dopamine and serotonin signals were obtained by performing *in vitro* liquid chromatography-tandem mass spectrometry on the harvested tissue. In the right dorsal hippocampal region, the gamma-aminobutyric-acid (GABA) and glutamine (Gln) concentrations were significantly higher in the sleep-perturbed rats than in the sham controls. The ratios of Gln/Glu (glutamate), Gln/tCr (total-creatine), and GABA/Glu were also significantly higher in the sleep-perturbed group, while serotonin concentrations were significantly lower in the sleep-perturbed rats. Pearson correlation results among individual rat data indicate that concentrations of dopamine (DA) and serotonin (5-HT) were significantly higher in SSP rats. A larger correlation coefficient was also observed for the SSP rats. Analysis of the correlation between the *in vivo* and *in vitro* signals indicated that the concentrations of Gln, 5-HT, and DA exhibited a significant negative correlation in the SSP rat data but not in that of control rats. The authors propose that the altered and correlated GABA, Gln, 5-HT, and DA concentrations/ratios could be considered key markers of neurological function in animal models of stress-induced sleep perturbation.

## Introduction

A stressful event can significantly influence sleep—wake behavior in all animals [1,2]. Stress-induced sleep perturbation (SSP) is a primary component of many pathophysiological conditions (i.e., those that cannot be attributed to psychiatric or medical disorders, substance abuse, or pain-related conditions), including post-traumatic stress disorder (PTSD) [2,3]. A secondary (comorbid) form can be observed in a wide variety of neurological, psychiatric, or medical conditions [3–5], such as insomnia [6].

Brady and Sinha reported that excessive exposure to stress is associated with alterations in the neurobiology of the brain and may lead to an increased risk of various psychiatric illnesses in humans, including PTSD [7]. Insomnia in PTSD is associated with an impairment of prolonged sleep, difficulties in continuing/maintaining sleep, reduced alertness, fatigue, and other symptoms [8,9]. The presence of an acute stressor can result in immediate disturbances such as increased wakefulness and inappropriate physiological arousal [1,2,4]. In addition, previous studies have suggested that sleep perturbation and loss of sleep cause significant, abnormal secretion of endogenous biomolecules [1,4,10]. Therefore, the present study will quantitatively assess the differences in neurochemical changes associated with sleep disturbance between sham controls and stress-induced models.

To date, numerous studies have shown that stress-induced rats exhibit significant metabolite abnormalities, functional impairments, and biological changes in both cortical and limbic regions, with particularly evident alterations in the prefrontal cortex [11,12]. Moreover, the hippocampus has been extensively investigated not only with regard to stress-related disorders, but also to cognitive, hormonal, and neurochemical actions [13,14]. Previous studies have suggested that acute and repeated exposure to stress results in increased expression of *c-Fos* mRNA, a marker of altered neuronal activity in the parvicellular subdivisions of the paraventricular hypothalamic nucleus (PVH) [2], as well as in the regions of the cortex, hippocampus, hypothalamus, septum, amygdala, and brainstem [2,15]. In particular, Melia et al. observed that *c-Fos* levels showed the greatest increases in the hippocampal region when compared to other regions, possibly due to changes in circulating levels of corticosterone [15]. The results of the aforementioned studies indicate that the hippocampus can be considered one of the most important areas with regard to neuronal activities associated with the stress response.

*In vivo* proton magnetic resonance spectroscopy (^1^H MRS) is a powerful and unique method that has enabled researchers to noninvasively quantify the cerebral neurochemical markers and neurotransmitters involved in neuro-molecular processes [16–18]. The utility of localized *in vivo* ^1^H MRS has been demonstrated in various studies of the brain and its disorders [19]. Cerebral neurochemical compounds contribute to the signal intensities and shapes of the various peaks on the *in vivo* ^1^H MR spectra [20] and can provide insight into which biomolecular compounds are implicated in neurological disorders [20]. Therefore, in order to obtain further knowledge of the biochemical compounds involved in SSP, analysis was performed using liquid chromatography-tandem mass spectrometry (LC-MS/MS). LC-MS/MS is a useful tool for assessing the *in vitro* status of metabolites and endogenous biomolecules from tissue, urine, blood, serum, and plasma [21]. LC-MS/MS also provides increased-resolution and signal-to-noise ratio (SNR), giving researchers the potential to identify the mechanisms relevant to selective brain pathologies [22]. In addition, the greater specificity, selectivity, and sensitivity of LC-MS/MS when compared with other methods of biochemical analysis has been studied and documented by researchers in various bioanalytical fields [23].

To the best of our knowledge, there have been no published reports regarding the cerebral metabolites and endogenous biomolecules implicated in stress-induced sleep perturbation in either human patients or rat models. Therefore, the goals of our study were to quantitatively assess (using *in vivo* ^1^H MRS and *in vitro* LC-MS/MS) the differences in these cerebral metabolites and to identify the factors that contribute to the alteration of endogenous biomolecules when rats undergo stress-induced sleep disturbance. We hypothesized that neurotransmitter signals would undergo alteration in the hippocampal region due to the neuronal dysfunctions and neurochemical abnormalities observed in sleep-deprived rats. We also hypothesized that the serotonin (5-HT: 5-hydroxytryptamine) and dopamine (DA) concentrations would differ between the hippocampal regions of the two groups because of the abnormal secretion of such endogenous molecules observed in sleep-deprived rats. In order to test these hypotheses, we compared the cerebral neurochemical responses and alterations of endogenous molecules in the stress-induced state between the sham control and sleep-deprived rats using *in vivo* ^1^H MRS and *in vitro* LC-MS/MS techniques.

## Materials and Methods

### Ethics statement

This study was carried out in strict accordance with the recommendations in the Guide for the Care and Use of Laboratory Animals of the National Institutes of Health. The protocol was approved by the University of Ulsan Animal Care and Use Committee (Permit Number: 2014-01-071). The stress induced sleep disturbance modeling was not fatal and *in vivo* MRI/MRS required mild anesthesia. The vitality of animals were checked before/after stress induced sleep disturbance modeling and the bio-signals (respiratory/heart rate) were monitored during *in vivo* MRI/MRS scan. Thus, no one was severely ill or died prior to our experimental endpoint. And all animals of our study have not received any other pains/stresses.

### Animals

Male Sprague-Dawley rats (eight weeks old, *n* = 20; weight = 270–315 g; Orient Bio, Pyeongtaek, Republic of Korea) were divided into two groups (sham-control group [CNTL]: *n* = 9; stress-induced, sleep-perturbation group [SSP]: *n* = 11). All animals were individually housed in standard plastic cages and maintained on a 12-h light—dark cycle (lights on at 08:00 A.M.) at an ambient temperature of 24.0–25.0°C, with ad libitum access to food and water.

### Stress-induced sleep perturbation (SSP)

Though previous studies utilizing sleep perturbation protocols have been published, the preset study focuses on the neurochemical profile observed during exposure of the SSP rat model to a psychological stressor [2]. Prior to initiating the experimental protocol, all rats were allowed free access to food and water for one week. Eleven SSP rats were placed in individual cages for one week without cage cleaning and were then placed into a dirty cage previously occupied by another male rat for one week (cage exchange). All SSP rats were left undisturbed in the previously occupied cage (dirty cage) until the start of the ^1^H MR spectra acquisition (5.5 hours after cage exchange). This time interval was chosen to reflect the previously established interval determined by Cano et al [2]. The study reported sleep fragmentation and gradually decreasing amounts of sleep beginning approximately 4 h after dirty cage exchange, while 1.5 h is the determined minimum time required for detection of neuronal activation (*Fos* expression) associated with a specific psychological stressor [2]. Nine CNTL rats were exchanged into a different set of clean cages at the same time (cage exchange at 11:00 A.M.) in order to synchronize the ultradian cycles between the two groups.

### *In vivo* ^1^H MRS at 9.4 T

Designs of the *in vivo* ^1^H MRS studies have previously been described [24]. All *in vivo* MR assessments were carried out using a horizontal 9.4 T/160 mm scanner (Agilent Technologies, Palo Alto, CA, USA), with 400 mT/m gradient sets. Prior to data acquisition, all rats were anesthetized using an isoflurane inhalation chamber at concentrations of 1.5–3.0%, and with a 5:5 mixture of N_2_O and O_2_ gas. The concentrations of the isoflurane anesthetic were maintained at 1–2% during MR data acquisition. All anesthetized rats were placed in the prone position with the head firmly fixed on a palate holder equipped with an adjustable nosecone and ear bars. All MR imaging and spectroscopy data were acquired using a 38 mm, 4-channel, quadrature, phased-array coil (for receipt), and a 72 mm volume coil (for transmitting). The animals breathed freely and maintained their body temperature during all MR assessments. Changes in respiratory rate were monitored in order to adjust the concentration of the anesthetic.

**Fig 1:**
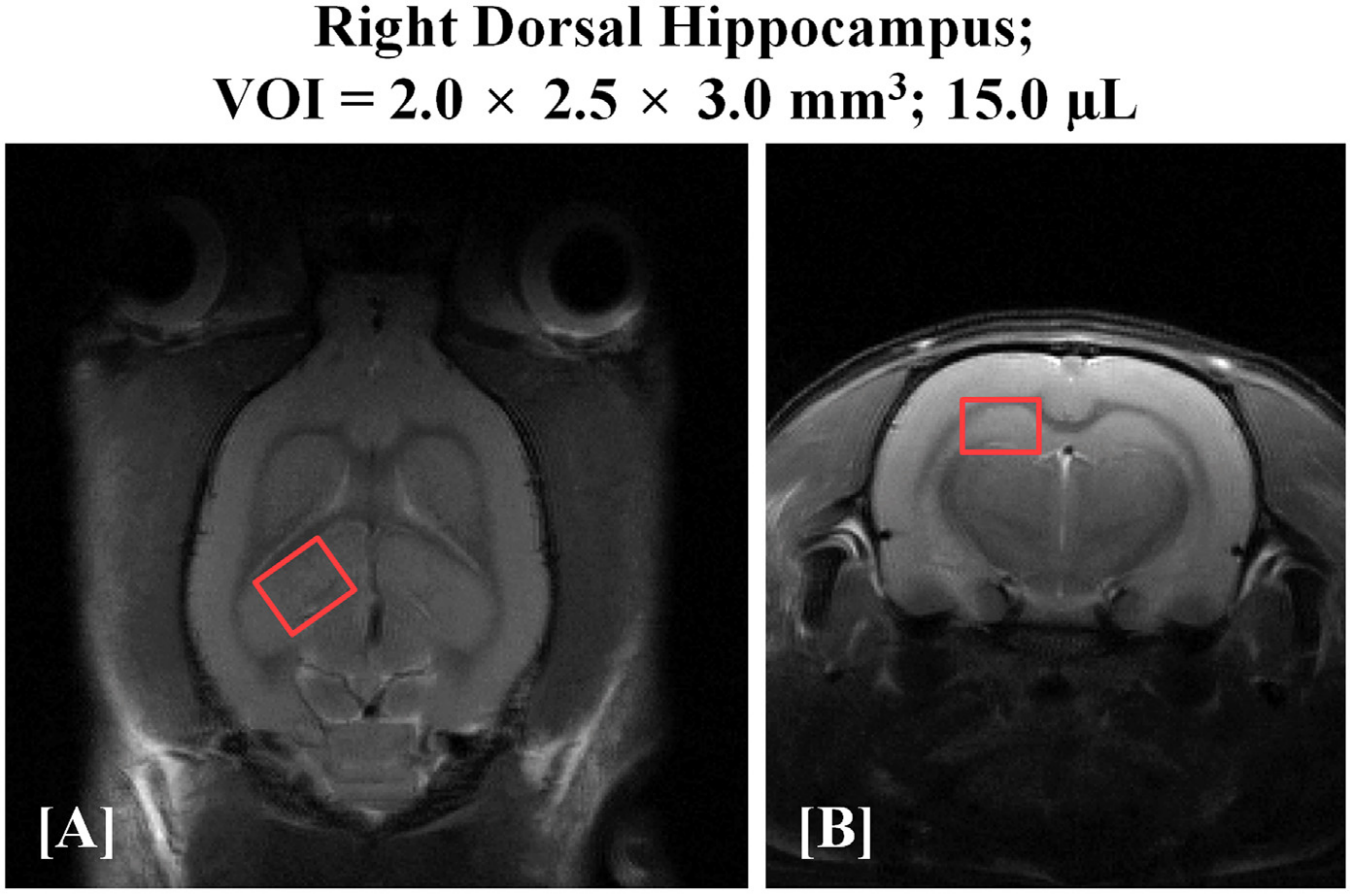
T2-weighted, FSE MR images [(A) coronal and (B) axial] of the rat brain with the volume of interest (VOI) centered in the right dorsal hippocampal region. The red color illustrations on T2-weighted images indicate the size of the rectangular volume of interest as 15.0 μL. *FSE*: *fast spin echo*.

For volume of interest (VOI) localization and identification of anatomical regions, multi-slice, T2-weighted MR images were acquired using a fast spin echo (FSE) pulse sequence (repetition time [TR] = 4000 ms, effective echo time [TE_eff_] = 32.95 ms, echo spacing [ESP] = 10.98 ms, echo train length [ETL] = 32, average = 1, field of view [FOV] = 30 × 30 mm, slice thickness = 1 mm, matrix size = 256 × 256, and total scan time = 2 min, 16 sec). According to the atlas of the rat brain [25], the VOI (2.0 × 2.5 × 3.0 = 15.0 μL) position was targeted to the right dorsal hippocampal region (Fig 1A and 1B), as indicated by the following coordinates: ± 1.0 mm at + 2.0 right of midline; ± 1.0–1.5 mm dorsal and ventral at interaural 7 mm; and ± 1.5 mm anterior and posterior at– 1.0 mm bregma. We carefully chose and adjusted the VOI position and size so as to minimize the inclusion of other anatomical regions and to avoid intracranial lipid contamination. Water suppressed *in vivo* ^1^H MRS spectra were acquired using a point-resolved spectroscopy (PRESS) pulse sequence with variable power and optimized relaxation delays (VAPOR) method (TR = 5000 ms, TE1/TE2/TE_total_ = 7.46/6.01/13.47 ms, spectral width = 5.0 kHz, average = 384, number of data points = 2048, and total scan time = 32 min 10 sec). Unsuppressed water spectra were acquired using the same parameters as those utilized to obtain water-suppressed spectra (except for averages and total scan time).

### Quantification of *in vivo* ^1^H MRS

Raw data acquired *in vivo* were analyzed using a fully blind spectral process, using Linear Combination Model software (LCModel, Version 6.2–1L and Copyright: Stephen W. Provencher) with a simulated basis set containing 18 metabolites, as follows: alanine (Ala); aspartate (Asp); creatine (Cr); phosphocreatine (PCr); gamma-aminobutyric acid (GABA); glutamine (Gln); glutamate (Glu); glucose (Glc); glycine (Glyc); glycerophosphocholine (GPC); scyllo-inositol (sI); myo-inositol (mIns); lactate (Lac); *N*-acetylaspartate (NAA); *N*-acetylaspartylglutamate (NAAG); phosphocholine (PCh); glutathione (GSH); and taurine (Tau). All signal intensities from the *in vivo* basis set were processed with water scaling and eddy current correction, and metabolite concentrations were obtained (μmol/g). All metabolites were fitted in the chemical shift range from 4.35 to 0.30 ppm.

### *In vitro* sample preparation

Following the completion of the *in vivo* ^1^H MRS assessment, all rats were sacrificed using greater than 95% carbon dioxide (CO_2_) gas. CO_2_ gas inhalation is a widely used, ethically acceptable method of euthanasia in rats due to rapid loss of consciousness at high concentrations (> 40%) [26]. In addition, intake of CO_2_ gas at high concentrations (> 70%) also results in narcotic effects on the brain without causing hypoxia [27,28]. Twenty, whole-brain tissue samples were quickly and carefully harvested from the removed skulls. Whole-brain tissue was immediately stored in liquid nitrogen (–196°C) in order to prevent biochemical changes and tissue decomposition.

The following compounds were purchased from Sigma-Aldrich or CDN Isotopes: DA hydrochloride; 5-HT hydrochloride; DA-1, 1, 2, 2-d4 hydrochloride; and 5-HT-α, α, β, β-d4 creatinine sulfate. Oasis WAX cartridges were obtained from Waters. All solvents, including water, were purchased from J. T. Baker. TissueLyzer (Qiagen) was used to homogenize 400–500 mg of cerebral hemisphere tissue in 400 μL of methanol. The homogenate was incubated for 15 min at 4°C. After sample incubation, 200 μL of 1 μM DA-1,1,2,2-d4 hydrochloride and 5-HT-α, α, β, β-d4 creatinine sulfate were added. The sample was then centrifuged at 14,000 rpm for 15 min. The supernatant was collected and an equal volume of 1.0% formic acid was added. The sample was mixed thoroughly and was then prepared for solid-phase extraction. A 3cc Oasis WAX cartridge was washed with 1 mL of methanol and conditioned with 0.5% formic acid sequentially. The sample solution was then loaded into the cartridge and incubated for 10 min. The cartridge was then completely dried using a vacuum. Finally, 1 mL of methanol was added for sample elution, and the eluent was dried under a vacuum. The dried sample was stored at– 20°C until analysis and reconstituted with 20 μL of 50.0% methanol prior to LC-MS/MS analysis.

### Quantification of *in vitro* LC-MS/MS

DA and 5-HT were analyzed using LC-MS/MS equipped with 1290 HPLC (Agilent Technologies), Qtrap 5500 (AB SCIEX), and a reverse phase column (Pursuit 5 C18 150 × 2 mm). Three μL of the solution were injected into the LC-MS/MS system and ionized with the turbo-spray ionization source. Formic acid in H_2_O (0.1%) and formic acid in methanol (0.1%) were used as mobile phases A and B, respectively. The separation gradient was as follows: hold at 10% B for 5 min; 10 to 70% B for 13 min; 70 to 90% B for 0.1 min; hold at 90% B for 8.9 min; 90 to 10% B for 0.1 min; and then hold at 10% B for 2.9 min. LC flow was 200 μL/min, and the column temperature was kept at 23°C. Multiple reaction monitoring (MRM) was used in the positive ion mode, and the extracted ion chromatogram (EIC) corresponding to the specific transition for each analysis was used for quantitation. The specific transitions (Q1/Q3) for DA and its internal standard were 154.1/137.1 and 158.1/141.1, while the specific transitions (Q1/Q3) for 5-HT and its internal standard were 176.9/160.0 and 181.0/164.0. The calibration range was 0.1–1000 nM with R^2^ > 0.98. Data analysis was performed using Analyst 1.5.2 software.

### Statistical analysis

All statistical analyses were performed using PASW Statistics 21 (SPSS Inc., IBM Company, Chicago, IL, USA). The present study evaluated the uncertainty of data fitting with regard to spectral analysis and only utilized values with Cramer-Rao lower bounds (CRLBs) exhibiting less than 30% standard deviation. All signal intensities from high-resolution spectra performed *in vivo* were processed with water scaling for quantification of metabolite concentrations and eddy current correction. Standard error estimates and CRLBs were used to provide estimates of reliability and uncertainty for each metabolite signal [29]. Metabolite concentrations with a CRLB of > 30% were regarded as not detected/validated. The CRLB values have been previously determine and established as reliable estimates for fitting uncertainty (LCModel and LCMgui User’s Manual; http://s-provencher.com/pages/lcm-manual.shtml) [30].

An independent t-test was used to compare the concentrations of the cerebral metabolites (17 signals) and endogenous biomolecules (5-HT and DA) in *in vivo* ^1^H MRS and *in vitro* LC-MS/MS. The mean values of the metabolite concentrations in SSP and control rats were respectively analyzed and compared to internal reference values using water signal. The statistically analyzed data are presented as mean values with positive standard deviations, unless otherwise indicated. Pearson correlations were used to analyze the relationship between the individual rat data of the two groups as well as the concentrations of cerebral metabolites and endogenous biomolecules. Probability (*p*) values less than 0.05 were considered statistically significant (* *p* < 0.05; ** *p* < 0.01; and *** *p* < 0.001).

The present study calculated the statistical power of the sample size using G*Power 3.1.9.2 software (http://www.phycho.uni-duesseldorf.de/abteilungen/aap/gpower3/). Initial values were set as follows: two tails, alpha error probabilities = 0.05, power = 0.8, allocation ratio = 1, and highest effect size = 1.003. The effect size was determined by the mean metabolite concentrations and standard deviation between two groups. Analysis using a sample size of 34 rats in two groups of revealed an actual power of 0.81.

## Results

### *In vivo* ^1^H MR spectra at 9.4 T

**Fig 2:**
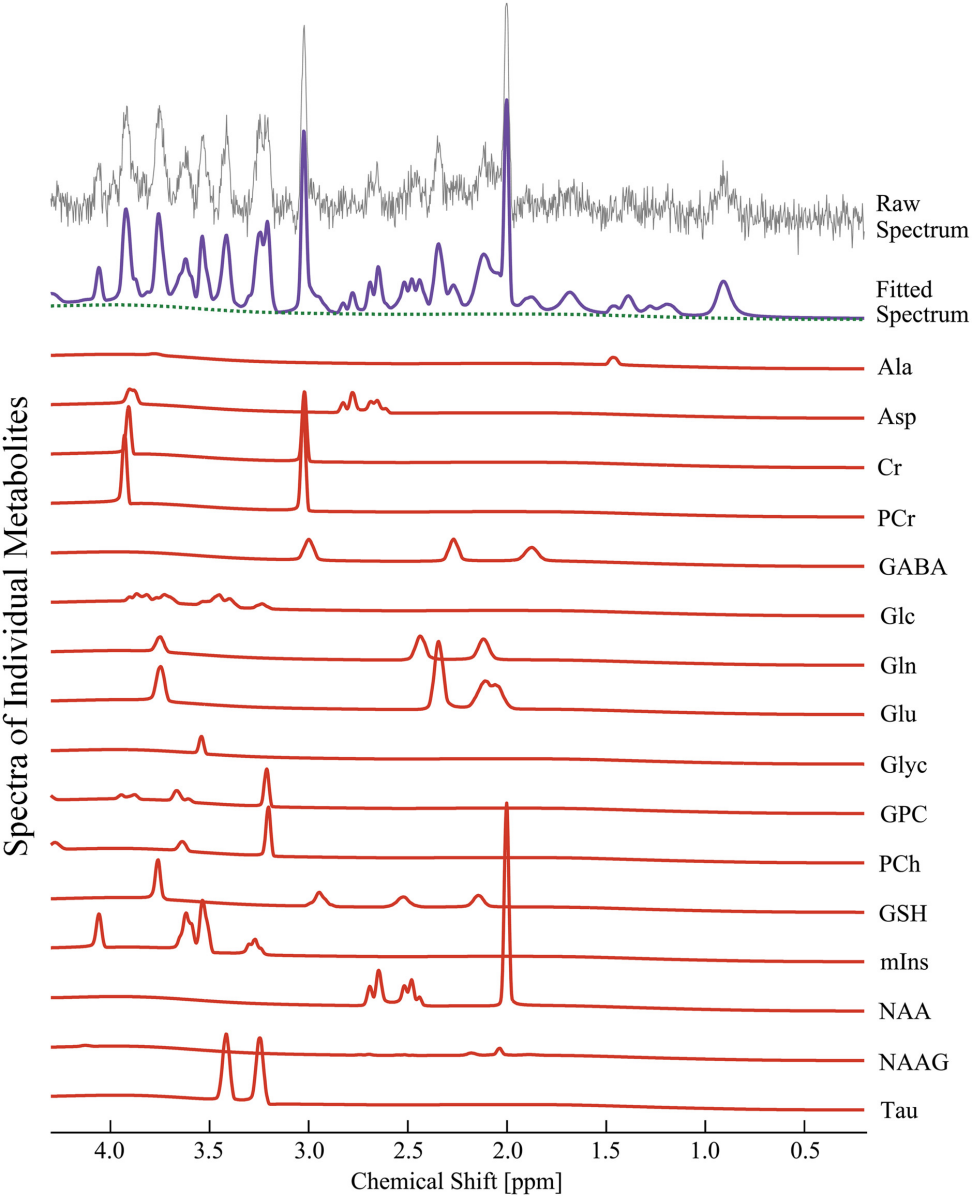
Quantification of 16 neuro-metabolite signals from *in vivo* ^1^H MRS in the right dorsal hippocampal region of the SSP model. The illustrations completely discriminated the raw spectrum (grey), fitted spectrum (purple), baseline (dotted green), and each neuro-metabolite signal (solid red) from the obtained *in vivo* ^1^H spectrum at 9.4 T. *SSP*: *stress-induced sleep perturbation*.

Fig 2 shows representative *in vivo* ^1^H MR spectra acquired from the right dorsal hippocampal region. All metabolite signals were quantified using LCModel, with a simulated basis set. The *in vivo* ^1^H MR spectra were assigned the following 17 cerebral neurochemical signals: Ala, Asp, Cr, PCr, GABA, Glc, Gln, Glu, Glyc, GPC, PCh, GSH, mIns, NAA, NAAG, Tau, and tCho (total choline). Fig 2 shows that the composition of each metabolite signal is visible in the narrow chemical shift ranges between 2.0–2.5 and 3.0–3.6 ppm. The CNTL spectra are not shown because visual inspection did not indicate any clear differentiation criteria compared to the SSP spectra. Sixteen metabolite signals from the *in vivo* ^1^H MR spectra were analyzed and observed to be within 30% percent of one standard deviation from the mean (% SD; CRLBs)

### Neurochemical changes in *in vivo* ^1^H MRS

**Fig 3:**
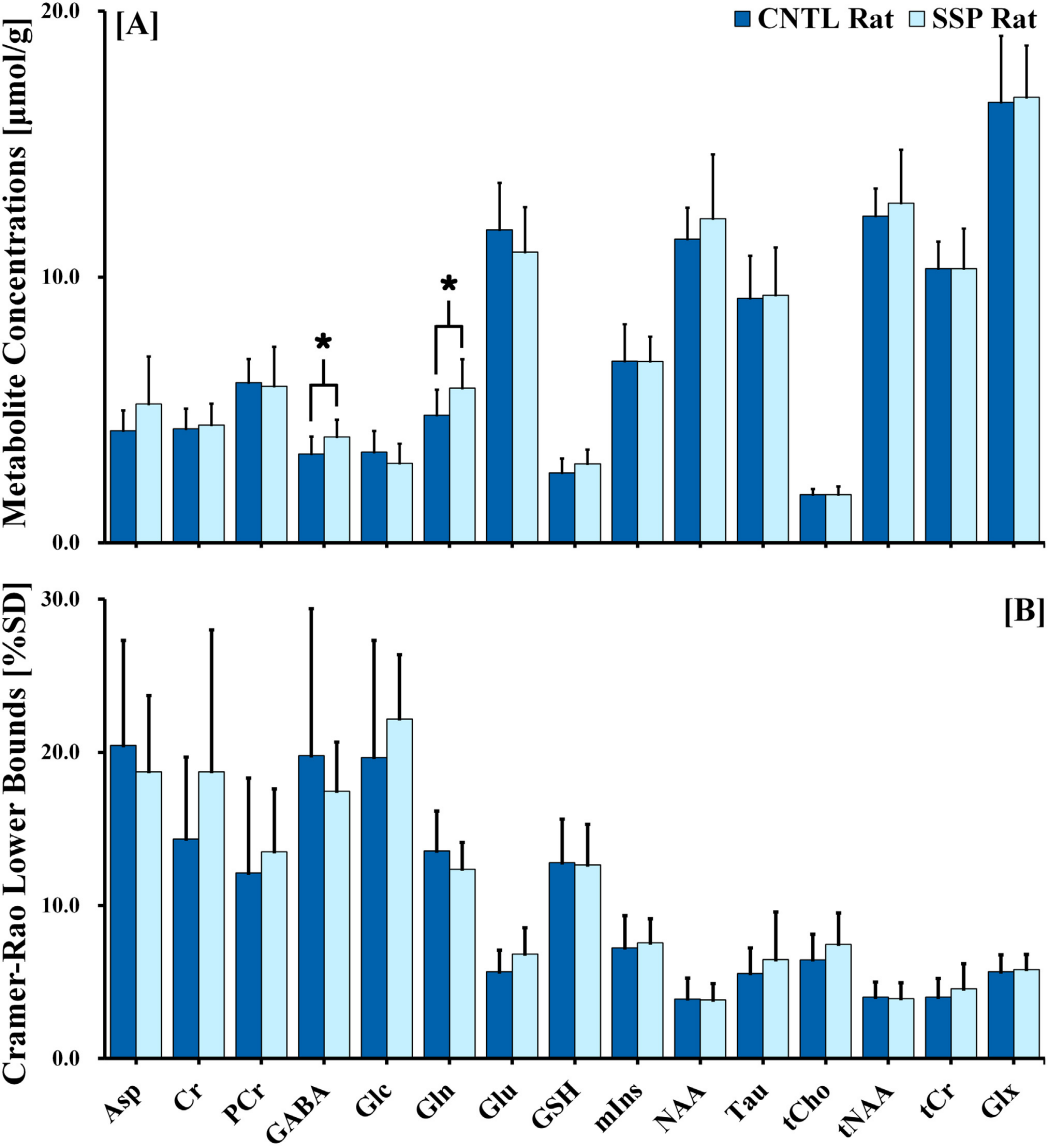
(A) Cerebral metabolite signals in the right dorsal hippocampal region of control and SSP rats, quantified from the LCModel fitted spectra. Metabolite concentrations are expressed as micromole per gram (μmol/g). Results were considered significant when independent t-tests revealed *p < 0.05. (B) The CRLBs are expressed in % SD. The vertical lines on each of the bars indicate the (+) standard deviation from the mean values of the metabolite concentrations. SSP: stress-induced sleep perturbation; CRLB: Cramer-Rao Lower Bound; % SD: percentage of standard deviation.

**Fig 4:**
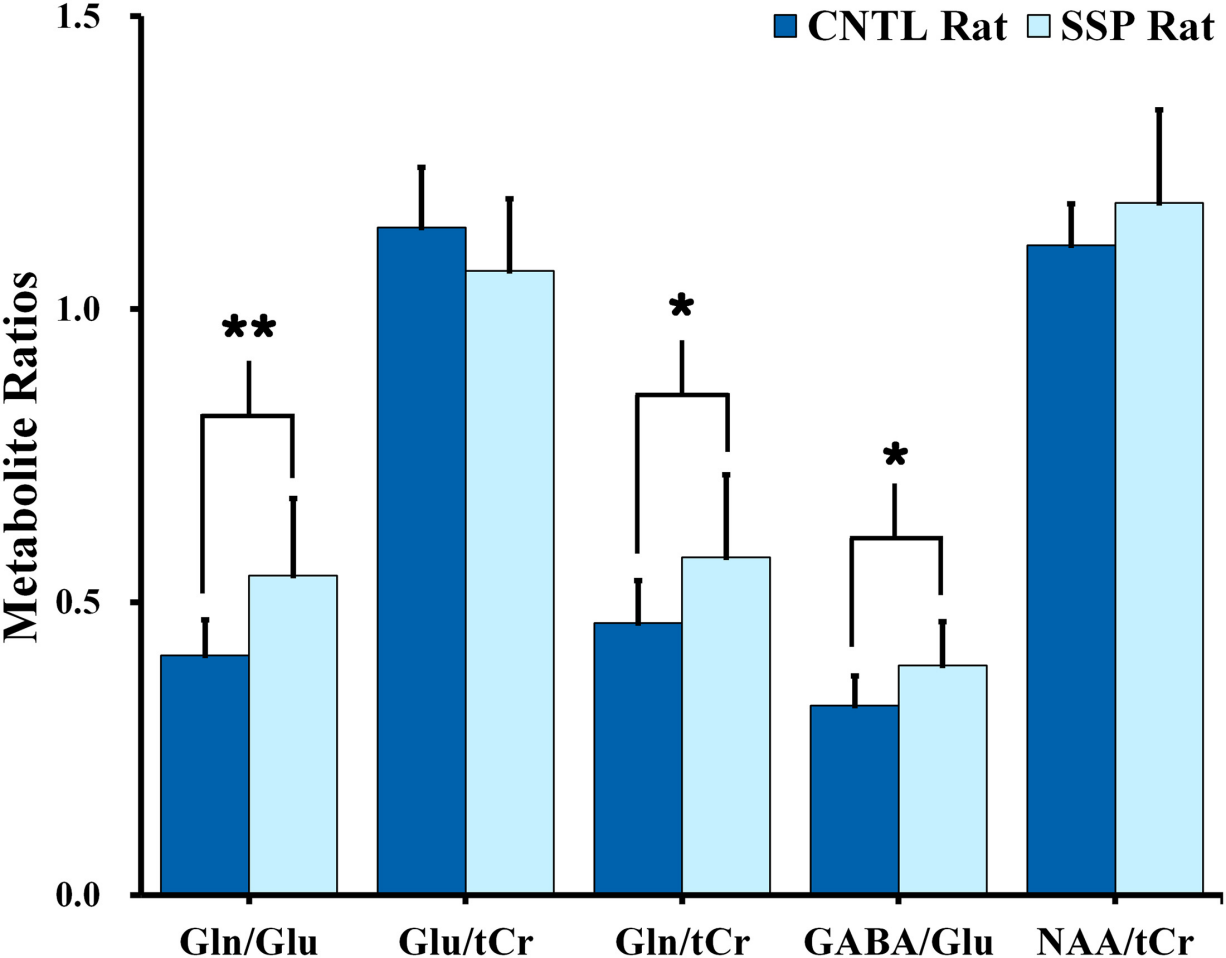
Bar graph illustrating the Gln/Glu, Glu/tCr, Gln/tCr, GABA/Glu, and tNAA/tCr ratios in the right dorsal hippocampal region of control and SSP rats. Results were considered significant when independent t-test revealed *p < 0.05, **p < 0.01. The vertical lines on each of the bars indicate the (+) standard deviation from the mean values of the metabolite concentrations. *Gln*: *glutamine; Glu*: *glutamate; tCr*: *total creatine; GABA*: *gamma-aminobutyric acid; tNAA*: *total N-acetylaspartate (NAA)*: *SSP*: *stress-induced sleep perturbation*.

Fig 3A and 3B illustrate the observed cerebral metabolite concentrations and the CRLB levels that were calculated from the acquired ^1^H MR spectra of the dorsal hippocampal region. An independent t-test revealed significant differences in the cerebral metabolite concentrations between the two groups, thus indicating a significant stress-induced effect on quantified metabolite concentrations. According to our results, concentrations of GABA (*F* = 0.040; df = 17.084; *p* = 0.040) and Gln (*F* = 0.357; df = 17.853; *p* = 0.038) were significantly higher in the SSP rats than in the CNTL rats. In addition, Fig 4 indicates that the Gln/Glu (*F* = 4.487; df = 14.844; *p* = 0.009), Gln/tCr (*F* = 5.985; df = 15.366; *p* = 0.037), and GABA/Glu (*F* = 0.322; df = 17.603; *p* = 0.025) ratios were significantly higher in the SSP rats than in the CNTL rats.

### Endogenous biomolecule changes in *in vitro* LC-MS/MS

**Fig 5:**
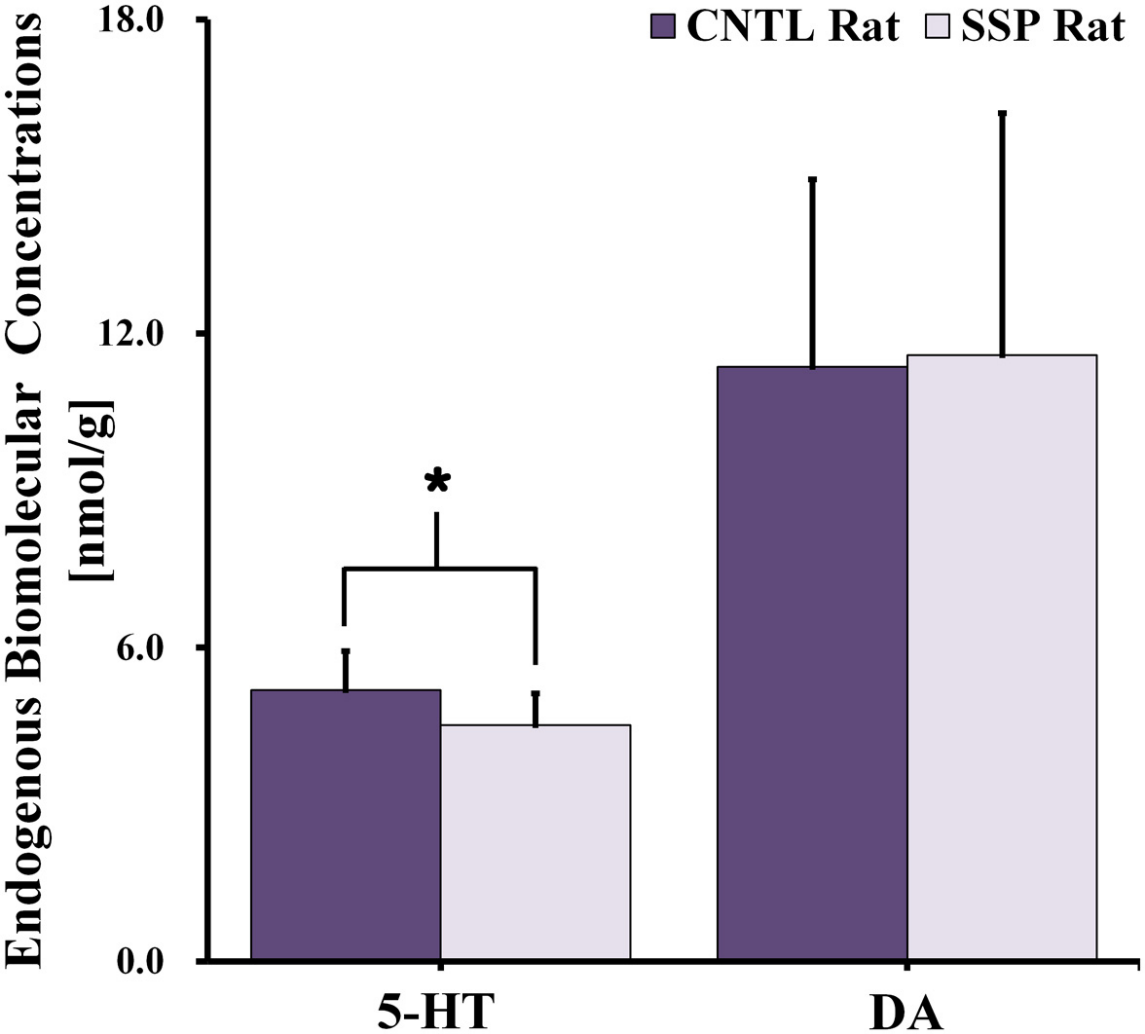
Endogenous biomolecule signals in the right dorsal hippocampal region of control and SSP rats, as quantified using LC-MS/MS. The concentrations in each signal are expressed as nanomole per gram (nmol/g). Results were considered significant when independent t-tests revealed *p < 0.05. The vertical lines on each of the bars indicate the (+) standard deviation from the mean values of the endogenous biomolecule signals. *SSP*: *stress-induced sleep perturbation; LC-MS/MS*: *liquid chromatography-tandem mass spectrometry*.

Fig 5 shows the 5-HT and DA concentrations that were quantified from the *in vitro* LC-MS/MS of twenty rat brain samples. Concentrations of 5-HT (*F* = 1.004; df = 15.402; *p* = 0.036) were significantly lower in the SSP rats than in the CNTL rats. However, our study indicated no significant difference in the DA concentration between the two groups.

### Neurobiological correlations of individual rat data

**Fig 6:**
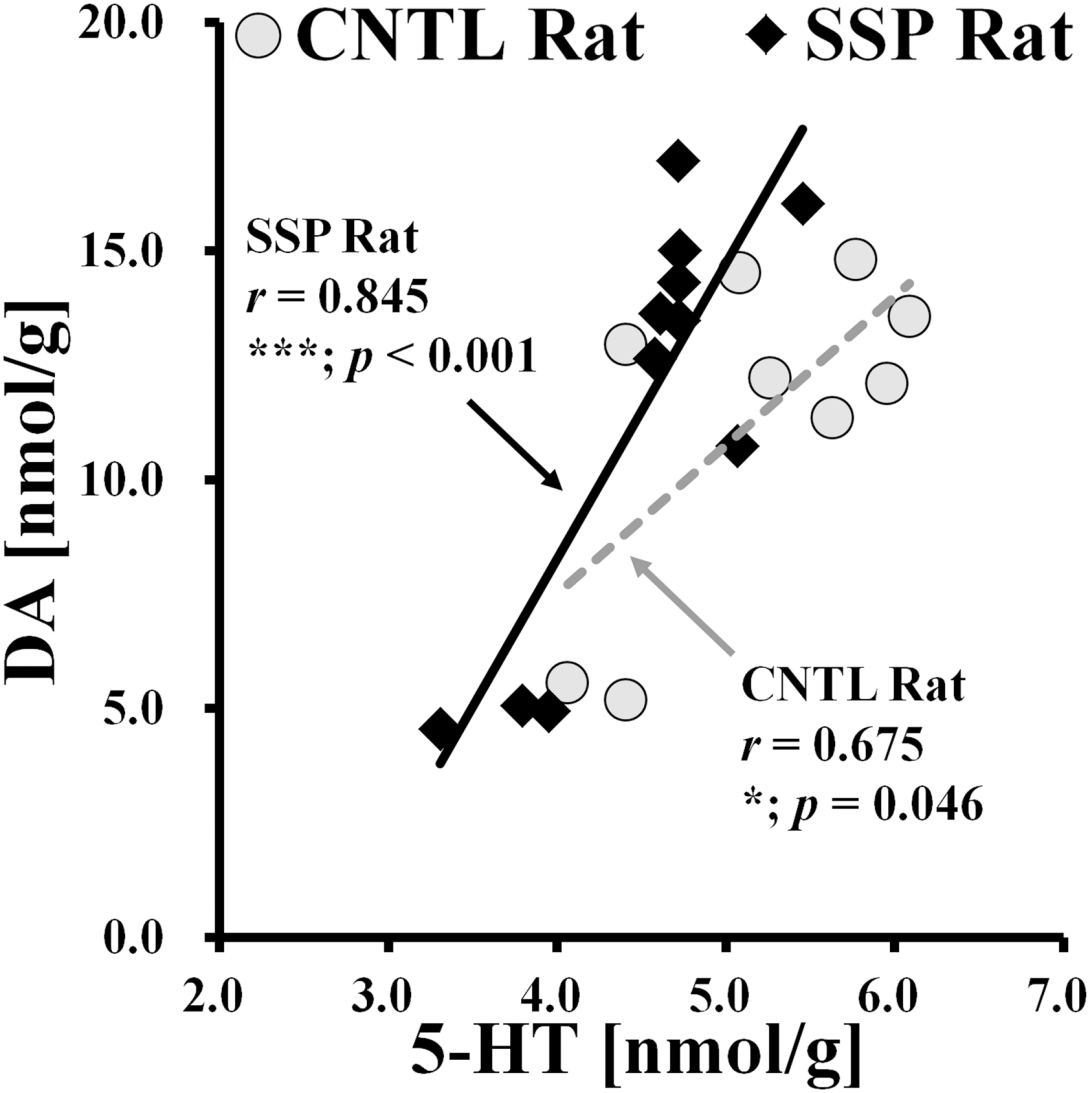
Scatter plots of signals obtained from endogenous biomolecules are quantified using data from individual rats. CNTL rats are represented with gray circles, while SSP rats are represented with black rhombi. Pearson correlation coefficients for individual clusters are represented as follows: CNTL (dotted line, gray) and SSP (solid line, black). The illustrations show the positive correlations of dopamine and serotonin concentrations between CNTL and SSP rats. The significance levels of the p values are as follows: *p < 0.05; ***p < 0.001. *DA*: *dopamine; 5-HT*: *5-hydroxytryptamine (serotonin)*.

In the present study, we determined that neurobiological fluctuations in metabolite and biomolecule concentrations in the SSP rat brain may exhibit significant linear associations. The results of the Pearson correlation indicated that concentrations of DA and 5-HT were significantly correlated among all individual clusters for both CNTL and SSP rats, in the right hippocampal region (Fig 6). Although linear scatter plots in each group clearly showed an increase in biomolecular concentrations, a higher level of significance (*p* < 0.001) and a larger correlation coefficient (*r* = 0.845) were observed for the SSP rats than for the CNTL rats.

**Fig 7:**
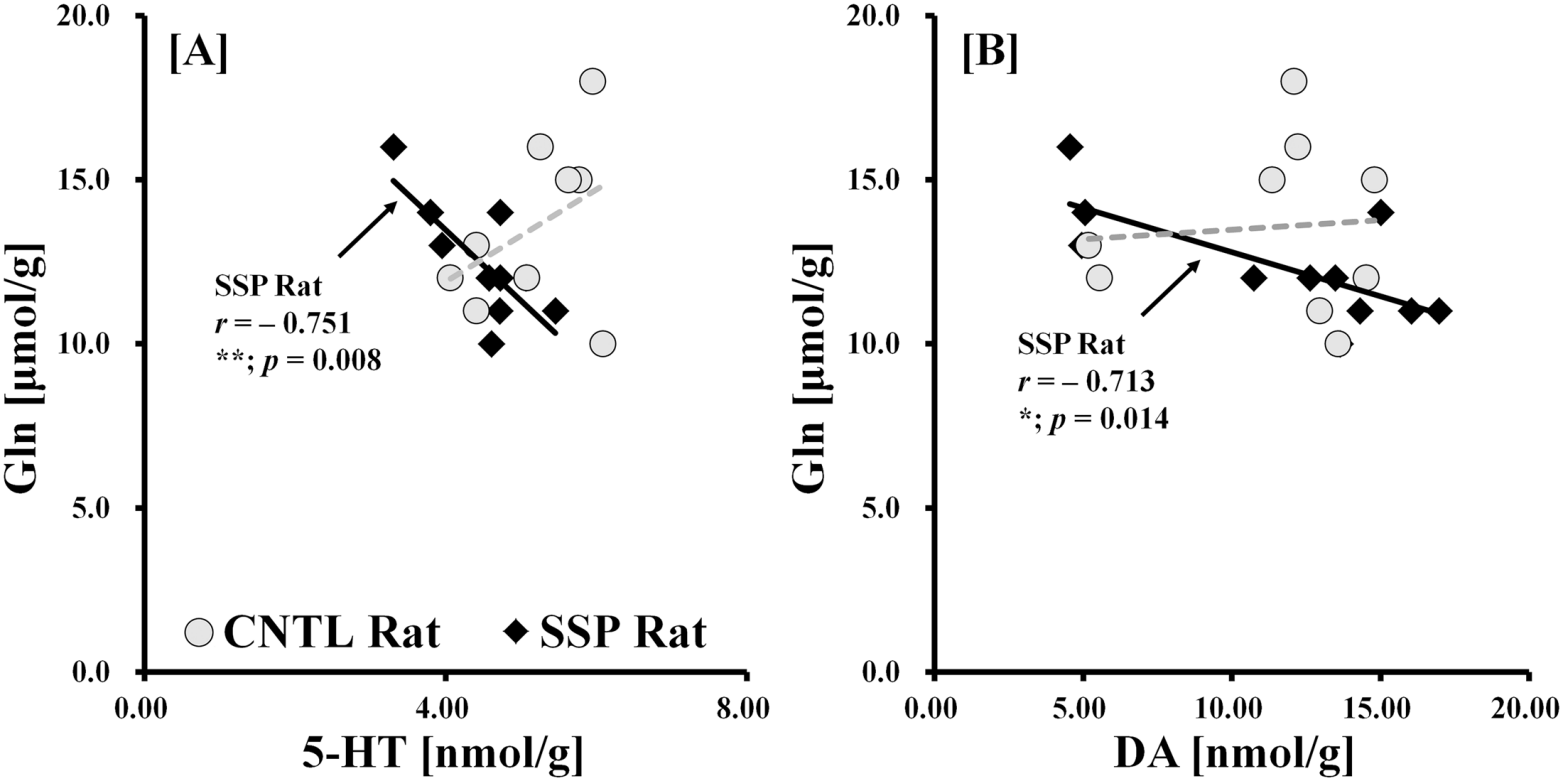
Scatter plots of the *in vivo* and *in vitro* signals quantified from individual rats. CNTL rats are represented with gray circles, while SSP rats are represented with black rhombi. Pearson correlation coefficients for individual clusters are represented as follows: CNTL (dotted line, gray) and SSP (solid line, black). The illustrations in A and B show the relationship between the *in vivo* and *in vitro* neurobiological concentrations as follows: Gln vs. 5-HT, and Gln vs. DA. The significance levels of the p values are as follows: *p < 0.05; **p < 0.01. *Gln*: *glutamine; DA*: *dopamine; 5-HT*: *5-hydroxytryptamine (serotonin)*.

To visualize the signal data obtained from metabolites *in vivo* and endogenous biomolecules *in vitro*, analysis was performed using linear scatter plots, which revealed a highly significant and reliable correlation (Fig 7A and 7B). The correlation results for Gln and 5-HT, and for Gln and DA concentrations were significantly negatively correlated in the SSP rat data [Gln vs. 5-HT (*r* = −0.751; *p* = 0.008); Gln vs. DA (*r* = −0.713; *p* = 0.014)] but not for that of CNTL rats [Gln vs. 5-HT (*r* = 0.401; *p* = 0.285); Gln vs. DA (r = 0.080; *p* = 0.838)]. In contrast to the results of the SSP correlations, data from the CNTL rats exhibited a positive relationship between *in vivo* and *in vitro* measurements.

## Discussion

To the best of our knowledge, our study is the first to use *in vivo* ^1^H MRS in combination with *in vitro* LC-MS/MS in a rat model to quantitatively assess the influences of stress-induced sleep perturbation on the cerebral neurochemical and endogenous biomolecular profile of the hippocampal region. The present study provides several new findings. First, based upon results obtained from *in vivo* ^1^H MRS, we observed that concentrations of GABA and Gln, as well as the ratios of Gln/Glu, Gln/tCr, and GABA/Glu, were significantly higher in the SSP rats than in the CNTL rats. Second, based upon results obtained from *in vitro* LC-MS/MS, concentrations of 5-HT were significantly lower in the SSP rats than in the CNTL rats. Lastly, we observed a correlation between the concentrations of DA and 5-HT, and *in vivo* and *in vitro* data.

Our study was based upon the potential relevance model with specific regard to insomnia, utilizing cage exchange as a sufficiently similar stressor. We hope that this similarity can be considered a starting point for assessing how such stressors affect sleep behavior [2].

### Stress-induced sleep perturbation modifies selective metabolite signals

Based on our results, GABA concentrations in the SSP rats were 19.4% higher than in the CNTL rats. However, in humans, previous studies have shown that patients suffering from primary insomnia exhibited significantly reduced GABA levels compared to healthy controls [3,31–33]. In addition, Morgan et al. suggested that this finding of significantly higher GABA levels in primary insomnia patients may be counterintuitive based on the current understanding of the role of GABA in sleep-wake behavior [34]. Significantly altered endogenous GABA levels may indicate that responses to a single acute stressor are associated with GABA_A_ receptor function [35,36]. Morgan et al. further suggested that GABA levels would gradually increase due to an allostatic response (neuro-adaptive response) caused by increased activity in the arousal system or inefficient GABA synthesis [34]. Therefore, we suspect that significantly elevated levels of GABA may reflect the relationship between GABA_A_ receptors and the time required to produce and regulate the appropriate stress response [34]. When the results of the current study are taken in conjunction with previous findings, the authors conclude that one possible explanation for such significantly higher levels of GABA might be that acute hyperarousal caused by stress-induced sleep perturbation results in an adaptive response that increases the functions of GABA_A_.

Assessments of the influences of stress-induced sleep perturbation on cerebral Gln and/or Glu concentrations are rare and reports are lacking. The present study observed significantly alterations in Gln and several related biomolecular ratios between the SSP rats and the CNTL rats. We found significantly higher Gln concentrations (21.2%) and Gln/tCr ratios (24.1%) following stress-induced sleep perturbation, which may point to alterations in the glutamate-glutamine (Glu-Gln) cycle [37] and reflect further alterations in glutamatergic turnover in the neuron-glia shuttle [37,38]. In addition, the Gln/Glu ratio was observed to be 33.2% higher in the SSP rats than in the CNTL rats. Glu and Gln are highly localized within glutamatergic neurons and astrocytes, respectively [39]. Glia provides a neuronal pathway for Glu synthesis and reuptake via the Glu-Gln cycle [40]. In particular, Moor et al. suggested that altered Gln metabolism might reflect the regulation of Gln-synthetase activities in the glia [40]. Therefore, significantly higher Gln concentrations may indicate an upregulation of Gln-synthetase activity, which could cause stress-induced sleep perturbation. Moreover, the significantly higher GABA/Glu ratios (21.1%) in SSP rats suggest that GABAergic activity is excessive while glutamatergic declines in models of sleep perturbation. We suggest that significantly higher Gln concentrations and Gln/Glu, Gln/tCr, and GABA/Glu ratios in SSP rats than those observed in CNTL rats may reflect the hyper-activity of glutamine synthetase and GABAergic receptors in the presence of declining glutamatergic activity. Our primary findings further suggest that GABA, Gln, and Glu signals in the hippocampal region are particularly sensitive and vulnerable to stress-induced sleep perturbation.

### Relevance of 5-HT and DA alteration

The results of the present study indicate that 5-HT concentrations were 13.0% lower in SSP rats than in CNTL rats. Although there were no significant differences in DA concentration between the two groups, a scatter plot revealed a positive correlation between DA and 5-HT based on the individual rat data. One major role of 5-HT is regulation of sleep-wake behavior [41–43]. L-Tryptophan is an essential amino acid, a precursor of 5-HT, and it is also implicated in the regulation of sleep [44]. In addition, the 5-HT precursors L-tryptophan and 5-hydroxytryptophane (5-HTP) have been reported to produce either increased length of total sleep or non-rapid eye movement sleep (NREM sleep) in rats [44,45]. Importantly, L-tryptophan and 5-HTP both play a functional role in the regulation of the total amount of sleep as well as in the various metabolic pathways involved [45]. Wyatt et al. reported that decreased levels of 5-HT resulted in reduced rapid eye movement sleep (REM sleep) and that increased levels of 5-HT resulted in elevated functioning of 5-HTP, causing an ultimate increase in the amount of REM sleep [46,47]. Therefore, based on these findings and the results of previous studies, significantly lower 5-HT concentrations in SSP rats than in CNTL rats might reflect a decrease in the rate of serotonin production, possibly due to dysfunctions or impairment in L-tryptophan and 5-HTP systems.

### Relationship between *in vivo*^1^H MRS and *in vitro* LC-MS/MS

Two significant neurobiological correlations were obtained from the *in vivo* and *in vitro* measurements. Analysis of the correlation results showed that Gln concentrations decreased significantly with increasing DA and 5-HT concentrations in the SSP rats, but not CNTL rats. Previous studies have identified changes in neurobiological concentrations such as elevated Gln [48,49] and reduced 5-HT and DA [49–52] in stress-induced models. Taken with these correlations, our results may indicate that Gln, 5-HT, and DA levels could function as key markers of neurological function in the stress-induced sleep disturbed rat brain. However, the influence of stress-induced sleep disturbance on cerebral metabolites and endogenous biomolecules has not been experimentally assessed with *in vivo* ^1^H MRS and *in vitro* LC-MS/MS, and the literature remains scarce. We cannot provide conclusive evidence in this regard because we did not experimentally assess the two types of *in vivo* and *in vitro* relationships, the neurobiological contributions, or the correlation among them in the stress-induced sleep disturbed rat model. Therefore, we conclude that further studies using pathological and neurophysiological assessments of the stress-induced states experienced during sleep disturbance are required to strengthen our conclusive findings and interpret the correlation among the various neurobiological signals.

### Limitations

There were some limitations to our methodology. First, as we focused on quantifying the alterations in the neurochemical profile induced by a psychological stressor in the region of the rat hippocampus, we did not assess the electroencephalogram (EEG) and electromyogram (EMG) using previously described surgical methodologies [2]. With regard to the surgical process previously described by Cano et al. [2], the implanted EMG electrodes (on nuchal) and pedestal sockets (on skull) of the rats may not be adjustable with the phased array coil. Therefore, further studies on stress-induced sleep perturbation using independent groups (for EEG/EMG measurement) are necessary in order to obtain a more quantitative assessment. Second, our study only analyzed metabolic changes in the right dorsal hippocampal region of the rats. Future studies should assess the metabolite changes across various brain regions. Third, several studies have reported that CO_2_ gas inhalation during euthanasia might cause hypoxia and hypercapnia, leading to breathlessness and hyperventilation [26,53]. Future studies should conduct quantitative assessments to determine the hypoxic influence of CO_2_ inhalation on the cerebral metabolites with various intake times and concentrations. Fourth, the present study did not measure anxiety-like behaviors or corticosteroid levels for validation of stress levels for considerable reasons: (1) As time was limited in order to obtain the most accurate model for stress-induced sleep disturbance, we chose to forego anxiety-like behavioral testing. We reasoned that obtaining *in vivo* measurements of neurochemical signals after 5.5 h was most important and that these signals and the sleep disturbed conditions could be affected by additional time delays and experiments. (2) We also reasoned that the animals may experience additional stress if we were to measure corticosteroid levels. Therefore, because we focused on quantifying the insomnia-related neurochemical changes induced by a psychological stressor, we did not conduct anxiety-like behavioral tests or measure corticosteroid levels. Further research is needed to obtain validation of our experimental results for both stress-induced sleep perturbed rats and control rats using various stress-related behavior tests, EEG, and corticosteroid measurement. Finally, the number of experimental rats we used was relatively small and may not provide conclusive evidence. Further studies based on larger populations are needed for a more definitive quantitative characterization of SSP-associated neurobiological responses.

## Conclusion

In summary, our study demonstrates that *in vivo* ^1^H MRS and *in vitro* LC-MS/MS data provide valuable information for interpreting the changes in the concentrations of specific cerebral metabolites and endogenous biomolecules in the hippocampal region of stress-induced, sleep-perturbed rats. Compared to the CNTL rats, there were significant alterations and correlations between Gln, GABA, 5-HT, and DA concentrations in the SSP rats. When taken in conjunction with the results of previous studies, our *in vivo* ^1^H MRS and *in vitro* LC-MS/MS results suggest that the GABA, Gln, 5-HT, and DA concentrations in the hippocampal region are particularly sensitive and vulnerable to stress-induced sleep perturbation. In addition, the present study proposes that the altered and correlated GABA, Gln, 5-HT, and DA concentrations/ratios could be considered key markers of neurological function in animal models of stress-induced sleep perturbation.

## References

[1] Bonnet MH, Arand DL. Hyperarousal and insomnia: State of the science. Sleep Med Rev. 2010; 14: 9–15. 10.1016/j.smrv.2009.05.002 19640748

[2] Cano G, Mochizuki T, Saper CB. Neural Circuitry of Stress-Induced Insomnia in Rats. J Neurosci. 2008; 28(40): 10167–10184. 10.1523/JNEUROSCI.1809-08.2008 18829974 PMC2693213

[3] Winkelman JW, Buxton OM, Jensen JE, Benson KL, O’Connor SP, Wang W, et al Reduced Brain GABA in Primary Insomnia: Preliminary Data from 4T Proton Magnetic Resonance Spectroscopy (1H-MRS). Sleep. 2008; 31(11): 1499–1506. 19014069 10.1093/sleep/31.11.1499 PMC2579978

[4] Lichstein KL, Wilson NM, Johnson CT. Psychological Treatment of Secondary Insomnia. Psychol Aging. 2000; 15(2): 232–240. 10879578 10.1037//0882-7974.15.2.232

[5] McCrae CS, Lichstein KL. Secondary insomnia: Diagnostic challenges and intervention opportunities. Sleep Med Rev. 2001; 5(1): 47–61. 12531044 10.1053/smrv.2000.0146

[6] Espie CA. Insomnia: Conceptual Issues in the Development, Persistence, and Treatment of Sleep Disorder in Adults. Annu Rev Psychol. 2002; 53: 215–243. 11752485 10.1146/annurev.psych.53.100901.135243

[7] Brady KT, Sinha R. Co-occurring mental and substance use disorders: the neurobiological effects of chronic stress. Am J Psychiat. 2005; 162: 1483–1493. 16055769 10.1176/appi.ajp.162.8.1483

[8] Pawlyk AC, Morrison AR, Ross RJ, Brennan FX. Stress-induced changes in sleep in rodents: Models and mechanisms. Neurosci Biobehav Rev. 2008; 32: 99–117. 17764741 10.1016/j.neubiorev.2007.06.001 PMC2215737

[9] Riemann D, Spiegelhalder K, Feige B, Voderholzer U, Berger M, Perlis M, et al The hyperarousal model of insomnia: A review of the concept and its evidence. Sleep Med Rev. 2010; 14: 19–31. 10.1016/j.smrv.2009.04.002 19481481

[10] Chrousos GP. Stress and disorders of the stress system. Nat Rev Endocrinol. 2009; 5: 374–381. 10.1038/nrendo.2009.106 19488073

[11] Gilad GM, Gilad VH, Wyatt W, Tizabi Y. Region-selective stress-induced increase of glutamate uptake and release in rat forebrain. Brain Res. 1990; 525: 335–338. 1979236 10.1016/0006-8993(90)90886-g

[12] Kim SY, Jang EJ, Hong KS, Lee C, Lee DW, Choi CB, et al Acute Restraint-Mediated Increases in Glutamate Levels in the Rat Brain: An *In vivo* ^1^H-MRS Study at 4.7 T Neurochem Res. 2012; 37:740–748. 10.1007/s11064-011-0668-y 22187117

[13] Bremner JD. Does Stress Damage the Brain? Biol Psychiatry. 1999; 45: 797–805. 10202566 10.1016/s0006-3223(99)00009-8

[14] Stewart MG, Davies HA, Sandi C, Kraev IV, Rogachevsky VV, Peddie CJ, et al Stress suppresses and learning induces plasticity in CA3 of rat hippocampus: a three-dimensional ultrastructural study of thorny excrescences and their postsynaptic densities. Neuroscience. 2005; 131(1): 43–54. 15680690 10.1016/j.neuroscience.2004.10.031

[15] Melia KR, Ryabinin AE, Schroeder R, Bloom FE, Wilson MC. Induction and Habituation of Immediate Early Gene Expression in Rat Brain by Acute and Repeated Restraint Stress. J Neuroscience. 1994; 14(10): 5929–5938. 7931554 10.1523/JNEUROSCI.14-10-05929.1994 PMC6576983

[16] Tkáč I, Gruetter R. Methodology of ^1^H NMR spectroscopy of the human brain at very high magnetic fields. Appl Magn Reson. 2005; 29: 139–157. 20179773 10.1007/BF03166960 PMC2825674

[17] Tkáč I, Henry PG, Andersen P, Keene CD, Low WC, Gruetter R. Highly Resolved *In vivo* ^1^H NMR Spectroscopy of the Mouse Brain at 9.4 T. Magn Reson Med. 2004; 52: 478–484. 15334565 10.1002/mrm.20184

[18] Lee DW, Kim SY, Kim JH, Lee T, Yoo C, Nam YK, et al Quantitative assessment of neurochemical changes in a rat model of long-term alcohol consumption as detected by in vivo and ex vivo proton nuclear magnetic resonance spectroscopy. Neurochem Int. 2013; 62: 502–509. 10.1016/j.neuint.2013.02.007 23411411

[19] Pfeuffer J, Tkáč I, Provencher SW, Gruetter R. Toward an *in vivo* neurochemical profile: Quantification of 18 metabolites in short-echo-time ^1^H NMR spectra of the rat brain. J Magn Reson. 1999; 141: 104–120. 10527748 10.1006/jmre.1999.1895

[20] Tzika AA, Cheng LL, Goumnerova L, Madsen JR, Zurakowski D, Astrakas LG, et al Biochemical characterization of pediatric brain tumors by using *in vivo* and ex vivo magnetic resonance spectroscopy. J Neurosurg. 2002; 96: 1023–1031. 12066902 10.3171/jns.2002.96.6.1023

[21] Jemal M. High-throughput quantitative bioanalysis by LC/MS/MS. Biomed Chromatogr. 2000; 14: 422–429. 11002279 10.1002/1099-0801(200010)14:6<422::AID-BMC25>3.0.CO;2-I

[22] Peng J, Elias JE, Thoreen CC, Licklider LJ, Gygi SP. Evaluation of Multidimensional Chromatography Coupled with Tandem Mass Spectrometry (LC/LC-MS/MS) for Large-Scale Protein Analysis: The Yeast Proteome. J Proteome Res. 2003; 2: 43–50. 12643542 10.1021/pr025556v

[23] Zhang G, Terry AV Jr., Bartlett MG. Sensitive liquid chromatography/tandem mass spectrometry method for the simultaneous determination of olanzapine, risperidone, 9-hydroxyrisperidone, clozapine, haloperidol and ziprasidone in rat brain tissue. J Chromatogr. 2007; B858: 276–281. 10.1016/j.jchromb.2007.08.007 PMC2697569 17766202

[24] Woo DC, Lenkinski RE. Neurochemical Changes Observed by *In vivo* Proton Magnetic Resonance Spectroscopy in the Mouse Brain Post administration of Scopolamine. Acad Radiol. 2014; 21: 1072–1077. 10.1016/j.acra.2014.04.003 25018079

[25] Paxinos G, Watson C. The Rat Brain in Stereotaxic Coordinates, 6th ed London: Elsevier Academic; 2007.

[26] Leach MC, Bowell VA, Allan TF, Morton DB. Aversion to Gaseous Euthanasia Agents in Rats and Mice. Comparative Med. 2002; 52(3): 249–257. 12102571

[27] Blackshaw JK, Fenwick DC, Beattie AW, Allan DJ. The behaviour of chickens, mice and rats during euthanasia with chloroform, carbon dioxide and ether. Lab Anim. 1988; 22: 67–75. 3127635 10.1258/002367788780746674

[28] Close B, Banister K, Baumans V, Bernoth EM, Bromage N, Bunyan J, et al Recommendations for euthanasia of experimental animals: Part 1. Lab Anim. 1996; 30: 293–316. 8938617 10.1258/002367796780739871

[29] Provencher S.W.. Automatic quantitation of localized *in vivo* ^1^H spectra with LCModel. NMR Biomed. 2001; 14: 260–264. 11410943 10.1002/nbm.698

[30] Fu Y., Serrai H.. Wavelet Encoding Spectroscopic Imaging in Small FOV Regime: Comparison to Chemical Shift Imaging at 3 Tesla. Appl Magn Reson. 2012; 43: 385–395.

[31] Kakeda S, Korogi Y, Moriya J, Ohnari N, Sato T, Ueno S, et al Influence of work shift on glutamic acid and gamma-aminobutyric acid (GABA): Evaluation with proton magnetic resonance spectroscopy at 3T. Psychiat Res. 2011; 192: 55–59. 10.1016/j.pscychresns.2010.10.011 21377845

[32] Levy LM, Degnan AJ. GABA-Based Evaluation of Neurologic Conditions: MR Spectroscopy. Am J Neuroradiol. 2013; 34: 259–265. 10.3174/ajnr.A2902 22268095 PMC7965110

[33] Plante DT, Jensen JE, Schoerning L, Winkelman JW. Reduced g-Aminobutyric Acid in Occipital and Anterior Cingulate Cortices in Primary Insomnia: a Link to Major Depressive Disorder? Neuropsychopharmacology. 2012; 37: 1548–1557. 10.1038/npp.2012.4 22318195 PMC3327859

[34] Morgan PT, Pace-Schott EF, Mason GF, Forselius E, Fasula M, Valentine GW, et al Cortical GABA Levels in Primary Insomnia. Sleep. 2012; 35(6): 807–814. 10.5665/sleep.1880 22654200 PMC3353043

[35] Acosta GB, Rubio MC. GABA_A_ receptors mediate the changes produced by stress on GABA function and locomotor activity. Neurosci Lett. 1994; 176: 291–231. 10.1016/0304-3940(94)90863-x 7970230

[36] Sanacora G, Mason GF, Rothman DL, Behar KL, Hyder F, Petroff OAC, et al Reduced cortical γ-aminobutyric acid levels in depressed patients determined by proton magnetic resonance spectroscopy. Arch Gen Psychiat. 1999; 56(11): 1043–1047. 10565505 10.1001/archpsyc.56.11.1043

[37] Broman J, Hassel B, Rinvik E, Ottersen OP. Biochemistry and anatomy of transmitter glutamate Handbook of chemical neuroanatomy. Amsterdam: Elsevier; 2000.

[38] Rothman DL, Sibson NR, Hyder F, Shen J, Behar KL, Shulman RG. *In vivo* nuclear magnetic resonance spectroscopy studies of the relationship between the glutamate-glutamine neurotransmitter cycle and functional neuroenergetics. Philos Trans R Soc B: Biol Sci. 1999; 354: 1165–1177. 10.1098/rstb.1999.0472 PMC1692640 10466144

[39] Rothman DL, Behar KL, Hyder F, Shulman RG. *In vivo* NMR studies of the glutamate neurotransmitter flux and neuroenergetics: Implications for brain function. Annu Rev Physiol. 2003; 65: 401–427. 12524459 10.1146/annurev.physiol.65.092101.142131

[40] Moor CM, Frazier JA, Glod CA, Breeze JL, Dieterich M, Finn CT, et al Glutamine and Glutamate Levels in Children and Adolescents With Bipolar Disorder: A 4.0-T Proton Magnetic Resonance Spectroscopy Study of the Anterior Cingulate Cortex. J Am Acad Child AdolescPsychiat. 2007; 46(4): 524–534. 10.1097/chi.0b013e31802f5f2c PMC4090056 17420688

[41] Deuschle M, Schredl M, Schilling C, Wüst S, Frank J, Witt SH, et al Association between a Serotonin Transporter Length Polymorphism and Primary Insomnia. Sleep. 2010; 33(3): 343–347. 20337192 10.1093/sleep/33.3.343 PMC2831428

[42] Monti JM. Serotonin control of sleep-wake behavior. Sleep Med Rev. 2011; 15: 269–281. 10.1016/j.smrv.2010.11.003 21459634

[43] Monti JM, Jantos H. The roles of dopamine and serotonin, and of their receptors, in regulating sleep and waking. Prog Brain Res. 2008; 172: 625–646. 10.1016/S0079-6123(08)00929-1 18772053

[44] Hartmann E, Chung R, Chien CP. L-Tryptophane and Sleep. Psychopharmacologia. 1971; 19: 114–127. 4935665 10.1007/BF00402635

[45] Hartmann E. The sleep-dream cycle and brain serotonin. Psychonom Sci. 1967; 8: 295–296.

[46] Jouvet M. Biogenic amines and the states of sleep. Science. 1969; 163(3862): 32–41. 4303225 10.1126/science.163.3862.32

[47] Wyatt R, Kupfer D, Sjoerdsma A, Engelman K, Fram D, Snyder F. Effects of L-tryptophan (A natural sedative) on human sleep. Lancet.1970; 296(7678): 842–846. 10.1016/s0140-6736(70)92015-5 4097755

[48] Zarate CA Jr., Du J, Quiroz J, Gray NA, Denicoff KD, Singh J, et al Regulation of cellular plasticity cascades in the pathophysiology and treatment of mood disorders. Ann N.Y. Acad Sci. 2003; 1003: 273–291. 14684452 10.1196/annals.1300.017

[49] Munck A, Guyre PM, Holbrook NJ. Physiological Functions of Glucocorticoids in Stress and Their Relation to Pharmacological Actions. Endocr Rev. 1984; 5: 25–44. 6368214 10.1210/edrv-5-1-25

[50] Mokler DJ, Torres OI, Galler JR, Morgane PJ. Stress-induced changes in extracellular dopamine and serotonin in the medial prefrontal cortex and dorsal hippocampus of prenatally malnourished rats. Brain Res. 2007; 1148: 226–233. 17368432 10.1016/j.brainres.2007.02.031 PMC2706085

[51] Machado RB, Tufik S, Suchecki D. Chronic stress during paradoxical sleep deprivation increases paradoxical sleep rebound: Association with prolactin plasma levels and brain serotonin content. Psychoneuroendocrinology. 2008; 33: 1211–1224. 10.1016/j.psyneuen.2008.06.007 18674865

[52] Bekris S, Antoniou K, Daskas S, Papadopoulou-Daifoti Z. Behavioural and neurochemical effects induced by chronic mild stress applied to two different rat strains. Behav Brain Res. 2005; 161: 45–59. 15904709 10.1016/j.bbr.2005.01.005

[53] Ludders JW, Schmidt RH, Dein FJ, Klein PN. Drowning is not euthanasia. Wildl Soc Bull. 1999; 27: 666–670.

